# Searching for the Majority: Algorithms of Voluntary Control

**DOI:** 10.1371/journal.pone.0003522

**Published:** 2008-10-24

**Authors:** Jin Fan, Kevin G. Guise, Xun Liu, Hongbin Wang

**Affiliations:** 1 Department of Psychiatry, Mount Sinai School of Medicine, New York, New York, United States of America; 2 Department of Neuroscience, Mount Sinai School of Medicine, New York, New York, United States of America; 3 School of Health Information Sciences, University of Texas Health Science Center at Houston, Houston, Texas, United States of America; Victoria University of Wellington, New Zealand

## Abstract

Voluntary control of information processing is crucial to allocate resources and prioritize the processes that are most important under a given situation; the algorithms underlying such control, however, are often not clear. We investigated possible algorithms of control for the performance of the majority function, in which participants searched for and identified one of two alternative categories (left or right pointing arrows) as composing the majority in each stimulus set. We manipulated the amount (set size of 1, 3, and 5) and content (ratio of left and right pointing arrows within a set) of the inputs to test competing hypotheses regarding mental operations for information processing. Using a novel measure based on computational load, we found that reaction time was best predicted by a grouping search algorithm as compared to alternative algorithms (i.e., exhaustive or self-terminating search). The grouping search algorithm involves sampling and resampling of the inputs before a decision is reached. These findings highlight the importance of investigating the implications of voluntary control via algorithms of mental operations.

## Introduction

The human body transmits 11 million bits of information per second (bps) to the brain, but our conscious mind can only process up to 50 bps (Information Theory, Britannica Online) [1]. For example, visual attention can select only 30 to 60 bits of information for processing with each glimpse [2]. Voluntary control [3] of information processing is therefore crucial to allocate resources and prioritize the processes so that those most relevant under a given situation can reach the level of focused consciousness. Although much of the reduction/selection (e.g., perceptual grouping [4]) has already occurred elsewhere and may be hard coded/wired, input information outside the current focus of attention cannot and should not be fully excluded because they may have survival value. Therefore, there is a need for dynamic and flexible control. Such control is also most needed when a great deal of computation is required prior to response generation, for example, during information processing in the presence of salient task-irrelevant distracters.

The mechanisms of voluntary control, however, are not well understood. Although studies on the neural correlates of voluntary control routinely employ tasks that manipulate control in a qualitative manner, e.g., the Erickson flanker task [5] or the color-word Stroop task [6], they have gathered important findings. Brain structures involved in selective sensory processing of relevant visual targets have also been studied using a cued spatial-attention task [7]. Further advance in our understanding of the specific roles of these structures, however, will come from a more quantitative investigation of the relationship between behavioral/neural responses and voluntary control. This requires parametrically examining the algorithms that instantiate the mental operations of voluntary control.

Searching for and identifying majority constituents of a group (e.g., if there are five children on a playground, three girls and two boys, then girls comprise the majority) is an important and common function of our daily lives. However, a hardwired circuit for the majority gate-based logic is inefficient to implement [8]. This may also apply to the human brain. Therefore, dynamic algorithms have to be employed for more flexible computations. In this study, we designed a majority function task to systematically manipulate the amount and content of input to examine the algorithms for the interplay of voluntary control with input. In this task, a set of 1, 3, or 5 horizontal arrows were presented simultaneously at 8 possible locations arranged as an octagon centered on a fixation cross. The ratio of left and right pointing arrows within a set was also manipulated. Participants were asked to determine the direction (left or right) in which the majority of arrows pointed, and to indicate their response via button press.

One way to quantify information is to measure its entropy. According to Shannon's information theory [9], the information uncertainty in bits (entropy) depends on the amount/content of the input and the efficiency of encoding. Therefore, we define computational load as entropy, which is determined by the information amount/content of the input and the algorithms of mental operations used to encode and process the input. Examination of changes in reaction time (RT) with respect to computational load allowed us to test competing hypotheses of the algorithms of mental operations that may be adopted by participants to control information processing to reach a majority decision.

## Methods

### Participants

Thirty adult volunteers participated in this study. After excluding six participants with a accuracy lower than 75% under the most difficult task condition, the final sample size included in this report is 24 (13 females and 11 males; mean age, 25.9 years; range, 22–38 years). Written informed consent was obtained from each participant following the procedure approved by the institutional review board of the Mount Sinai School of Medicine. All participants had normal or corrected-to-normal vision.

### Apparatus and procedures

The task was compiled and run on a PC with a 17 inch LCD monitor, using E-Prime™ software (Psychology Software Tools, Pittsburgh, PA). The task was first explained using a paperboard illustrating each condition. Participants then performed a practice session on a PC with 6 blocks of trials with 12 trials in each block and 72 trials in total, using the same timing parameters as the actual test. The practice was continued until participants demonstrated at least 90% accuracy overall. Participants then performed the actual test.

### The majority function task (MFT)

**Figure 1:**
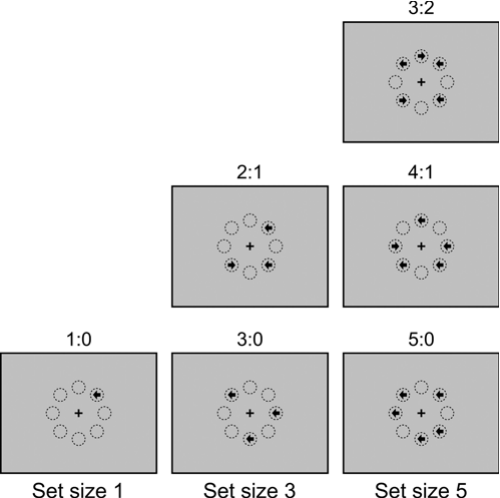
Illustration of representative stimulus configurations of the majority function task. In this task, arrows with set sizes of 1, 3, or 5 are randomly presented at 8 possible locations arranged as an octagon centered on a fixation cross. The arrows point either left or right, and are presented simultaneously. Participants' task is to indicate the direction in which the majority of arrows point. For example, if three arrows are presented, and two point to the left and one to the right (see the “2∶1” panel in the “Set size 3” column), the correct response should be “left”. The eight circles are for illustration of the locations and are not displayed during the experiment. The label for each condition is the ratio of the numbers in each category.

In this task, groups of arrows with set sizes of 1, 3, and 5 are randomly presented at 8 possible locations arranged as an octagon centered on a fixation cross. The arrows point either left or right, and are presented simultaneously (see **Figure 1**). The configuration of the 8 positions is similar to that used in previous studies on covert attention [10], [11]. On a computer screen, the length of the arrow is 6 mm, the radius from the fixation cross to the center of any arrow is 2 cm, and the viewing distance is 50 cm. The radius from the fixation cross to the center of an arrow subtends approximately 2.3° of visual angle. Participants' task is to indicate the direction in which the majority of the arrows point. To encourage speed without sacrificing accuracy, participants are instructed to make responses as rapidly as possible while maintaining a low error rate. In each trial, an arrow set is presented for 2500 ms, followed by a variable fixation period of 2000 to 3000 ms. Each trial lasts 5 s on average. Responses within the 2500 ms window terminate the display of the stimulus. There are three runs in this task. In each run, there are two blocks for each set size, six blocks in total. Each block has 12 trials. Within a block for a certain set size, arrows under different stimulus conditions are displayed in a random order, with each stimulus condition appearing an equal number of times. The order of the blocks is counterbalanced by a Latin square with reversed repetition within each run. The order is 135-531, 513-315, and 351-153 for the first, second, and third run, respectively. Here the number represents the set size. The total number of trials in each run is 72. Before and after each block there is a 5 s fixation period. There are also five 5 s fixation periods between blocks in each run. Each run lasts 395 s. The total trial number in this task is 216 and the task takes about 20 minutes.

Although the amount and content of input to be processed is varied in the majority function task, the response is only one bit because there are only two alternatives. Therefore, the variable related to the stages of response selection and execution, after stimulus preprocessing and categorization [12], is constant across all set sizes and stimulus conditions. In addition, the pattern presented in a current trial is independent of its preceding trial, in contrast to the serial-choice RT tasks.

## Results

### Behavioral results

**Table 1** shows the experimental results including mean RT and accuracy under each condition. Although it is possible to infer the computational load of each condition from the accuracy of the responses [13], here we used RT as the main dependent variable. The mean RTs (520 ms, 884 ms, 1200 ms) for the three set sizes were significantly different, F(1, 23) = 792.18, p<0.01 (linear), and F(1, 23) = 2.69, ns (quadratic). In set size 3, the RTs under the two conditions were significantly different, F(1, 23) = 608.19, p<0.01. In set size 5, the RTs under the three conditions were significantly different, F(1, 23) = 813.95, p<0.01 (linear), and F(1, 23) = 25.09, p<0.01 (quadratic). The mean accuracy (99.5%, 98.7%, 94.6%) of the three set sizes were significantly different, F (1, 23) = 96.22, p<0.01 (linear), and F(1, 23) = 21.39, p<0.01 (quadratic). In set size 3, the accuracy under the two conditions were significantly different, F(1, 23) = 10.12, p<0.01. In set size 5, the accuracy under the three conditions were significantly different, F(1, 23) = 109.07, p<0.01 (linear), and F(1, 23) = 69.23, p<0.01 (quadratic). The positive change in RT, mean standard deviation, and error rate across conditions may represent the differences in the computational load.

**Table 1:** RT (ms) and accuracy (%) under all stimulus conditions (n = 24).

Set size	Stimulus condition	Ratio	RT			Accuracy	
			Mean	SD	Mean SD^a^	Mean	SD
1	0,1	1∶0	520	77	107	99.5	0.9
3	000, 111	3∶0	647	110	142	100.0	0.0
	001, 011	2∶1	1121	153	309	97.5	3.9
5	00000, 11111	5∶0	724	130	174	99.8	0.9
	00001, 01111	4∶1	1261	192	349	98.6	2.4
	00011, 00111	3∶2	1615	203	392	85.2	6.7

Note: ^a^ Mean of SDs across participants.

### Analysis of algorithms of mental operations

The behavioral results suggest a relationship between RT and amount/content of inputs that goes beyond a simple linear or loglinear function, suggesting an interaction between uncertainty of inputs and mental operations in overall performance. We calculated computational load as a function of input information to be processed and the algorithms adopted by the human brain. Therefore, potential algorithms have to be compared and contrasted in order to find the most plausible one. Methods used in the analysis of short-term memory scan (e.g., [14]) can be used to analyze the algorithm of mental operations. Here we analyzed and compared the results using three plausible algorithms: exhaustive search, self-terminating search, and grouping search.

#### Exhaustive search

If we follow the equation to find the majority (the majority function), which takes all inputs and then returns the value which is most common among them, we would expect that, for all stimulus conditions within the same set size, computational load and RT would not be affected by the number of arrows pointing in a common direction. That is, the processing time for this algorithm is only affected by the amount, and not by content, of input. For example, for a set size of 5 arrows, RT would be the same for conditions in which 5, 4, and 3 arrows are pointing in the same direction. The data indicated that this was not the case.

#### Self-terminating search

Given that arrows are presented in random patterns and locations, assuming that human participants scan the arrows sequentially and terminate the scan when the majority of the arrows can be determined, we can compute the computational load in terms of bits under different input conditions. Let 0 and 1 represent left and right pointing directions, respectively. For set size 1, there are only two possible outcomes: 0 or 1. For set size 3, there are four combinations: 000, 001, 011, 111 (disregarding the order of the digits in each combination). For the set size 5, there are six combinations: 00000, 00001, 00011, 00111, 01111, and 11111. For set size 1, only one arrow with two alternatives has to be scanned. Therefore the computational load in bits is 1. For set size 3, if three arrows point in the same direction (000, or 111), only two arrows need to be scanned. Therefore, the computational load in bits is 2. However, if only two arrows point in the same direction, corresponding to the three patterns of 001, 010, and 100 (considering the order), there will be 2, 3, or 3 arrows that need to be scanned sequentially starting from the left and moving to the right. The same number of bits applies to the combinations of stimulus condition 011. On average 2 2/3 arrows need to be scanned. Therefore, the average computational load in bits is 2 2/3, with the best-case of 2 and worst-case of 3. Similarly, for set size 5, having 5, 4, or 3 arrows pointing in the same direction, the average the computational load in bits is 3, 3 3/5, and 4 1/2, with the best-case being 3 for all three conditions, and worst-case being 3, 4, and 5 for each of the three conditions, respectively (see **Table 2**).

**Table 2:** Experimental conditions and estimates of input information and computational load under self-terminating and grouping search algorithms.

Set size	Stimulus condition	Ratio	Input digits	Self-terminating search			Grouping search			
				Best	Worst	Average	Group a	Group size	Scan digits	log_2_
1	0,1	1∶0	1	1	1	1	1	1	1	0.00
3	000, 111	3∶0	3	2	2	2	1	2	2	1.00
	001, 011	2∶1	3	2	3	2 2/3	3	2	6	2.58
5	00000, 11111	5∶0	5	3	3	3	1	3	3	1.58
	00001, 01111	4∶1	5	3	4	3 3/5	2.5	3	7.5	2.91
	00011, 00111	3∶2	5	3	5	4 1/2	10	3	30	4.91

Note: ^a^ Number of grouping attempts on average required to obtain a congruent sample.

**Figure 2:**
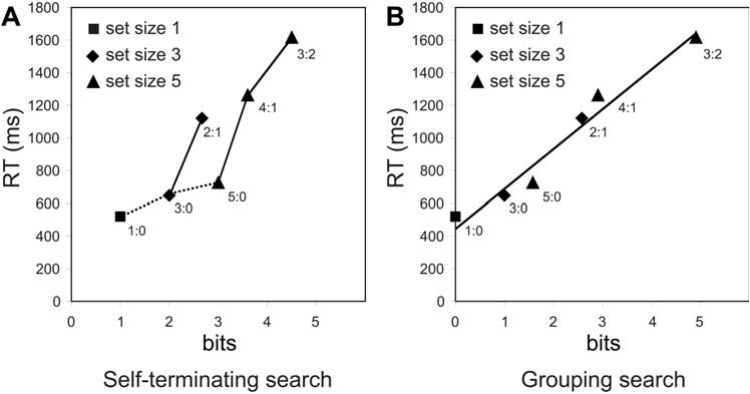
Reaction time (RT) as a function of computational load which is determined by processed information in bits, on average, assuming that the self-terminating search algorithm was adopted (A), and that the grouping search algorithm was adopted (B). The grouping search algorithm better predicts the linear relationship between the RTs and computational load relative to the self-terminating search algorithm.

**Figure 2A** shows a plot of the mean RT as a function of computational load in terms of bits assuming the self-terminating search algorithm was used. The regression analysis with RT as the dependent variable and computational load in bits as the independent variable was conducted. For the average case, RT = 110+312 · bits, R^2^ = 0.82, F(1, 4) = 17.73, p<0.05, indicating a good fit. Linear mixed-effects model analysis with computational load as the fixed effect and subject as the random effect showed that Akaike's information criterion (AIC) was 1945.06; the intercept was significant, F(1, 131) = 4.88, p<0.05; and the computational load was significant, F(1, 119) = 423.33, p<0.01. However, the RT of the incongruent condition of set size 3 (2∶1 condition, with a computational load of 2 2/3 bits) was significantly longer than the congruent condition of set size 5 (5∶0 condition, with a computational load of 3 bits), 1121 vs. 724, t(23) = 6.08, p<0.001. Given that the self-terminating algorithm predicts less computational load in the former condition than the latter, this evidence is against the self-terminating search and suggests that participants may have adopted strategies beyond the self-terminating search to perform the task.

#### Grouping search

The algorithm implemented in the human brain may not be as simple as the above-mentioned self-terminating search. When human participants analyze patterns in order to make a response, they may adopt a strategy in which they attempt to group and sample arrows with a majority size (over half of the total set size) based on their directions in order to achieve a high efficiency. For example, for a set size of 3, intuitively, a Boolean circuit of (*a_1_* XNOR *a_2_*) OR (*a_1_* XNOR *a_3_*) OR (*a_2_* XNOR *a_3_*) makes sense. Here the exclusive nor (XNOR) returns “true” if input *a_1_* and *a_2_* are identical, and returns “false” if they are different. If the grouping of (*a_1_* XNOR *a_2_*) returns true, the search can stop. However, this grouping (or sampling) process may have to be performed several times independently or recursively before a solution is reached based on a congruent sample. Therefore, more computation would be required under near-tie high uncertainty conditions within a certain set size than what would have been predicted based only on the self-terminating search algorithm. It is noteworthy to indicate that the maximum grouping size of 3 arrows should be within the capacity limit of the locations that can be selected at once [15].

Let us assume that participants adopt such a grouping (sampling) strategy and search for a congruent sample with a majority grouping size. For set size 1, only 1 arrow needs to be scanned. For set size 3, for the condition in which all 3 arrows point in the same direction, only 1 grouping attempt needs to be made with 2 arrows being scanned; and for the condition in which 2 arrows point in the same direction, there will be 1 successful grouping out of an average of 3 attempts. Therefore, 6 arrows, the product of 3 grouping attempts and group size of 2 arrows, need to be scanned. Similarly, for set size 5, for the conditions in which 5, 4, or 3 arrows point in the same direction, 1, 2.5, 10 grouping attempts on average need to be made and 3, 7.5, and 30 arrows need to be scanned, respectively (see **Table 2**).

If we use the majority group size (1, 2, and 3 for set sizes of 1, 3, and 5, respectively) as the information unit, assuming that each sampled group is equivalent to one unit of information, the information to be processed (i.e., the computational load) is log_g_ (s), where the base g represents the group size and s is the number of arrows to be scanned. To convert this measure to bits (i.e., from base g to base 2), it is multiplied by log_2_ (g) [9]. Therefore, the computational load is log_2_ (g) • log_g_ (s), which is equivalent to log_2_ (s). Here we converted the information to be processed in each condition to bits based on the average number of arrows that need to be sampled. It is worth noting that the calculation of 0 bits for the set size 1 condition does not mean that 0 bits of information need to be processed. The decision making step of the majority direction needs 1 bit. We can add 1 bit to all conditions, but this should not affect the general predictions of the grouping search algorithm.

**Figure 2B** depicts RT as a function of computational load assuming the grouping search algorithm was adopted. The regression analysis with RT as a function of computational load of the grouping algorithm was also conducted on the group data. With the log_2_ (scan arrows) (i.e., computational load in bits) as the independent variable, RT = 458+242 · bits, R^2^ = 0.96, F(1, 4) = 105.48, p<0.001. Linear mixed-effects model analysis with computational load as the fixed effect and subject as the random effect showed that AIC was 1823.29; the intercept was significant, F(1, 38) = 246.36, p<0.01; and the computational load was significant, F(1, 119) = 1398.48, p<0.01. Compared to the self-terminating search model, the grouping search model fits the data better because the AIC value of the linear mixed-effects model for the grouping search was lower than for the self-terminating search.

#### Self-report of the strategy adopted by participants

Participants were queried regarding the strategy that they each employed during the task at the end of the study. Of 24 subjects, 17 reported that they scanned the stimulus display until they found either 2 arrows pointing in the same direction (for set size 3) or found 3 arrows pointing in the same direction (for set size 5). Eight of these subjects spontaneously mentioned that they found the task to be the easiest when 2 or 3 arrows pointing in the same direction were grouped together, and the remainder agreed that the task was the easiest when this occurred when prompted by the experimenter. Six participants spontaneously described use of a grouping strategy. For example, one participant reported, for a set size of 5 arrows, first identifying a group of three arrows. If all arrows in the group pointed in the same direction, the participant made the appropriate response. If only two of the three did, the participant scanned the rest of the display for a third arrow pointing in the same direction. If grouping was not possible, e.g. the arrows were evenly distributed about the crosshair, then a serial scanning strategy was adopted.

## Discussion

Other than exhaustive search, self-terminating search, which incorporates an additional stopping rule in which the participant scans the arrows one by one until the majority threshold is reached (e.g., 2 arrows pointing in the same direction within a set size of 3 arrows), is clearly the second most straightforward algorithm. Consistent with this algorithm, Figure 2A reveals that RT increases with the computational load in two cases: (a) across the three congruent conditions, in which all arrow(s) in each set point in the same direction, as indicated by the dashed line; and (b) within the two conditions of set size 3 and within the three conditions of set size 5, as indicated by the solid lines. However, the opposite prediction for the 2∶1 and 5∶0 conditions based on self-terminating search stands as evidence against the possibility that subjects adopted this algorithm.

The fact that human visual attention can be directed towards more than one item simultaneously allows for the possibility of a grouping search algorithm, in which participants first select a sample of arrows with a size equal to the majority threshold and then process the sample. This is similar to perceptual grouping [4]. If all arrows in the sample happen to point in the same direction (congruent), then a response can be quickly generated. If not, a re-sampling kicks in until a congruent sample is found and a response is generated. We estimate the computational load for the grouping search as a logarithmic function of the product of the grouping size and the expected number of groups that need to be sampled in order to obtain one that is congruent. It is clear that RT increases monotonically as a function of the computational load and that this relationship is well approximated by a linear function (Figure 2B). The linear relationship between RTs and the computational load based on the grouping search strategy may suggest a tree-like structure representing the arrows to be sampled and a dichotomizing test.

These results support the idea that RT is determined not only by the amount and content of the input but also by the algorithms of mental operations that people adopt in the face of information uncertainty. Situations in the real world are often more complex than laboratory choice-RT tasks and require more voluntary control. The majority function task is interesting in that it requires greater voluntary control of computation than tasks used for testing the conflict effect (e.g., [5]), although it also uses conflicting information to manipulate information uncertainty. With this task we highlight the role of voluntary control during information processing and provide a more general framework to account for the conflict effect. For example, in a variation of the flanker task [16] in which people were asked to detect the direction of the target arrow and ignore the distracters, we observed a typical conflict effect–the RT difference between the incongruent and congruent conditions–of about 50–150 ms. This can be accounted for by the computational load framework because the computational load for the congruent condition is 1 bit for the two alternative responses, whereas it is less than or equal to 2 bit for the incongruent condition because of incongruent flankers.

In addition, performance in the majority function task cannot be fully predicted by a conflict effect account. For example, comparing two conflicting conditions in which the distribution of arrows are 2∶1 and 4∶1 in set sizes 3 and 5 respectively, the RT of the latter condition is significantly longer (1121 vs. 1261, t(23) = 4.55, p<0.001) , which is opposite of what the conflict effect account predicts, since the non-target to target ratio is larger in the former. Similar to other categorization tasks [12], the goal of computation is to identify the majority based on the input. Because any arrow could potentially belong to the majority subset if the set size is equal to or greater than 3, more than one arrow needs to be processed, either scanned one-by-one or randomly sampled and grouped as we tested above. However, the degree of uncertainty caused by conflicting information predicts RT within a given set size if the grouping search is adopted. For example, for the set size 5, the most uncertain condition with the distribution of arrows of 3∶2 requires 10 grouping attempts to be made on average before a solution is reached based on the grouping search algorithm. This may explain why its RT is much longer (1615 ms) compared to another less uncertain but also conflicting condition with the distribution of arrows of 4∶1 (1261 ms), which requires only 2.5 attempts on average to obtain a congruent sample.

The majority function task reported in this paper has features of conflict, grouping, and input variation that are often elements of many separate tasks in the literature. The methods to compute the computational load in this task may be used to account for discrepancy between findings of previous studies on conflict effect using different tasks. This majority function task is similar to the visual motion task used in studies of perceptual decision making (e.g., [17]), but here we examine and model decision making on a system level by considering the algorithms potentially adopted by the brain to process discrete information. We cannot exclude other possible factors that might contribute to the current results such as the Gestalt effect based on the holistic perception of all congruent arrows, information reduction [18], or perceptual grouping [4]. This may account for overall faster responses and the relatively flat slope for the congruent conditions. Although certain common mechanisms might be involved in voluntary control, the underlying algorithms will vary and be task specific in different situations depending on different computational goals [19]. For example, under the high input information condition, which is beyond the grouping capacity limit (e.g., more than 5 arrows), other algorithms, such as those suggested for perceptual decision making regarding motion coherence, might be adopted by humans to find the majority.

We argue that voluntary control is implemented by algorithms of mental operations, which are in turn implemented by brain networks. This study demonstrates that it is important and plausible to analyze the underlying algorithms for voluntary control by examining the relationship between the amount and content of input and RT. RT is a basic and central measure of mental operations in almost all cognitive tasks [20]. Early studies based on information theory [9] have found that choice RT is determined by the amount of information in bits that has to be processed to generate a correct response (e.g., [18], [21], [22]), though the causality in this relationship has been challenged [23], [24]. Some elegant models for the central mechanisms of choice RT have been proposed, and changes in RT as a function of information processing have been studied in the context of perceptual decision making (e.g., the sequential-sampling models, for a review, see ref. [25]), mental addition (subtraction) [26], visual search [27], and categorization [28]. In this study, we explicitly considered the underlying algorithms for voluntary control of information processing.
